# Tactile Sensing and Control of Robotic Manipulator Integrating Fiber Bragg Grating Strain-Sensor

**DOI:** 10.3389/fnbot.2019.00008

**Published:** 2019-04-05

**Authors:** Luca Massari, Calogero M. Oddo, Edoardo Sinibaldi, Renaud Detry, Joseph Bowkett, Kalind C. Carpenter

**Affiliations:** ^1^Polo Sant'Anna Valdera, The BioRobotics Institute, Scuola Superiore Sant'Anna, Pontedera, Italy; ^2^Department of Linguistics and Comparative Cultural Studies, Ca' Foscari University of Venice, Venice, Italy; ^3^Center for Micro-BioRobotics, Istituto Italiano di Tecnologia (IIT), Pontedera, Italy; ^4^Jet Propulsion Laboratory, California Institute of Technology, NASA, Pasadena, CA, United States; ^5^Department of Mechanical and Civil Engineering, California Institute of Technology, Pasadena, CA, United States

**Keywords:** robotics, manipulation tasks, fiber bragg gratings, tactile sensors, sensorized hand

## Abstract

Tactile sensing is an instrumental modality of robotic manipulation, as it provides information that is not accessible via remote sensors such as cameras or lidars. Touch is particularly crucial in unstructured environments, where the robot's internal representation of manipulated objects is uncertain. In this study we present the sensorization of an existing artificial hand, with the aim to achieve fine control of robotic limbs and perception of object's physical properties. Tactile feedback is conveyed by means of a soft sensor integrated at the fingertip of a robotic hand. The sensor consists of an optical fiber, housing Fiber Bragg Gratings (FBGs) transducers, embedded into a soft polymeric material integrated on a rigid hand. Through several tasks involving grasps of different objects in various conditions, the ability of the system to acquire information is assessed. Results show that a classifier based on the sensor outputs of the robotic hand is capable of accurately detecting both size and rigidity of the operated objects (99.36 and 100% accuracy, respectively). Furthermore, the outputs provide evidence of the ability to grab fragile objects without breakage or slippage e and to perform dynamic manipulative tasks, that involve the adaptation of fingers position based on the grasped objects' condition.

## Introduction

**Figure 1:**
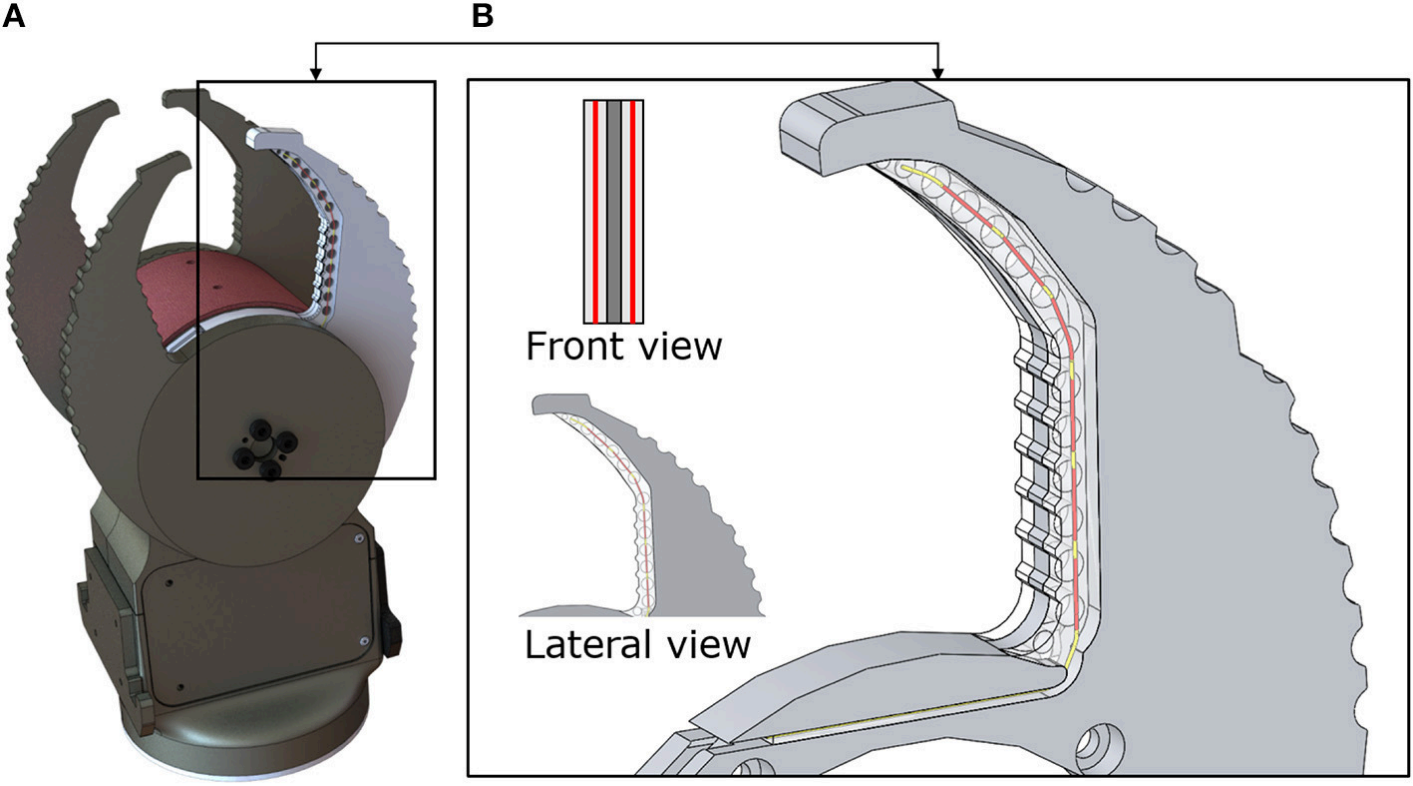
**(A)** Cam-Hand. **(B)** Inset of the sensorized finger. The red lines represent the FBGs. Each optical fiber houses 6 FBGs (8 mm length).

The sense of touch is a key sensory modality of prehensile manipulation. Through tactile perception, humans can perceive object properties such as size, hardness, temperature, contour, etc. Information arises from the multiple receptors available within the human skin, especially across hand and fingers (Johansson and Vallbo, 1979; Johansson et al., 1982). During manipulation, the hand partially occludes the object from sight. Tactile sensing enables measurements to be obtained in areas that are inaccessible through vision. Prior behavioral studies have demonstrated the tactile reliance of human manipulation, for both simple grasping and dexterous manipulation (Johansson and Flanagan, 2009). In the last few years, the field of robotics has expanded toward more complex environments (Dahiya et al., 2010), including dangerous and unaccessible scenarios such as nuclear meltdown disasters and space missions to other planets, where robots are demanded to take over human jobs. The successful automation of complex human-like manipulative tasks depends on robot's perception capabilities, including through a tactile sensor, to characterize the relation between the operated objects and the robotic manipulator (Tegin and Wikander, 2005; Hoshi and Shinoda, 2006; Yousef et al., 2011). Although the human hand represents a point of inspiration for many prehensile robotic hardware (Bicchi, 2000; Murray, 2017), the field of artificial tactile sensing covers a large spectrum of underlying principles (Chi et al., 2018). The literature, for instance, shows relative success with capacitive, piezoelectric, piezoresistive, and resistive sensors. Such sensing systems rely on changes in the measured variable (i.e., capacitance, electrical charge, resistance, etc.) that involve different advantages and disadvantages. Capacitive sensors consist of two conductive plates interfaced by means of a compressible dielectric material (Golpaygani et al., 2009; Wong et al., 2012; Vogt et al., 2013). The transduction principle relies on the capacitance variations that occur when, during the loading phase, the gap between the plates changes. Such transducers entail high sensitivity and frequency response but are susceptible to electro-magnetic noise, tend to be non-linear and to have hysteresis. Capacitive sensors are extensively used in robotic applications for tactile feedback, (Romano et al., 2011; Schmitz et al., 2011; Heyneman and Cutkosky, 2012; Jara et al., 2014). Piezoelectric sensors depend on the electrical charge generation in the quartz crystal, as it deforms by applying a load. Such sensors are frequently employed for dynamic sensing, due to a very high frequency response and can be used to build flexible tactile sensors (Sirohi and Chopra, 2000; Cutkosky et al., 2008; Qasaimeh et al., 2009; Chuang et al., 2013; Seminara et al., 2013; Canavese et al., 2014; Kim et al., 2014; Acer et al., 2015). On the other hand, piezoelectric sensors suffer temperature sensitivity and are generally fragile (Dahiya and Valle, 2008). Piezoresistive sensors rely upon the electrical changes in resistance occurring to the material during load/pressure application (Girão et al., 2013; Ma et al., 2015; Oddo et al., 2016). Such sensors are widely used as they are relatively easy to produce and can be flexible (Someya and Sekitani, 2014). The main drawbacks of these transducers refer to the low repeatability, fragility to shear forces, non-linear response and hysteresis. Among all the technologies, the use of optical fibers as transducers for tactile sensors is spreading due to the multiple advantages such as: electromagnetic immunity, flexibility, high sensitivity, multiplexing capability, and lightness (Polygerinos et al., 2010; Udd and Spillman, 2011; Wang and Wolfbeis, 2012). Several studies promote such sensors for different fields of application such as: automotive (da Silva et al., 2010), medicine (Silvestri and Schena, 2011) and smart textile (Massaroni et al., 2015) among the others. Depending on the working principle, fiber optic based sensors entail different ways of operation: micro and macro bending (Heo et al., 2007; Pirozzi, 2012), interferometry (Liu et al., 2012), hybrid optoelectronics (Ascari et al., 2007) and Fiber Bragg Grating (FBG) (Liang et al., 2018). In parallel to the development of tactile sensors, the robotics community has produced a vast amount of research on hand design. Hand design is typically application-driven, leading to different arrangements ranging from simple two-finger grippers to complex contraptions that mimic the mechanics of the human hand (Eusebi et al., 1994; Ramos et al., 1999; Townsend, 2000; Butterfaß et al., 2001). This paper presents the case of a four-finger under-actuated hand (Cam-Hand) that endows Jet Propulsion Laboratory's (JPL) quadruped RoboSimian robot with both manipulation and versatile mobility capabilities (Hebert et al., 2015; Karumanchi et al., 2018). This robot uses its limbs for mobility and manipulation such as grasping. Each seven degree of freedom limb consists of a set of three elbow assemblies and an actuator mechanically linked to the main body. The limbs end with a six-axis force sensor which interfaces the Cam-Hand (Figure 1A). The hand consists of an aluminum body and four aluminum fingers configured for many uses including being used as a gripper tool. The chosen design, conceived for use in scenarios that require robust manipulation, resulted in a system that enhances grip strength and robustness over dexterity and flexibility. Not being designed to prioritize complex manipulation tasks or handling fragile objects limits the variety of tasks the robot is able to perform. The present work is aimed at overcoming these limitations and enhancing safety and control during interaction with the surrounding environment. The RoboSimian Cam-Hand has been redesigned by sensorizing the artificial fingers to enable tactile feedback. New sensorized robotic fingers have been devised, embedding optical fiber sensing technologies, to gain information on grasped object properties as well as the contact conditions. The choice of the robotic hand sensorization was based on some crucial requirements such as (i) the ability to provide information about the contact (i.e., intensity), (ii) the ability to provide information about the grasped objects (i.e., size, rigidity, etc.), and (iii) the ability to perform manipulation tasks (i.e., estimation of grasp stability) (Kappassov et al., 2015). Moreover, it is worth mentioning that the robotic hand presents additional constraints related to the physical integration of the sensors. The adopted technology has to meet the requirements imposed by the robotic hand layout and design. Hence, the sensorization needs to be achieved without affecting the dexterity of the hand, i.e., without drawbacks in terms of bulkiness and encumbrance. Considering the aforementioned physical and tactile requirements, FBG technology was chosen to realize the sensor due to its adaptability to the design of the artificial hand, for its reliability in strain measurements and for the multiplexing capabilities that entail a high spatial resolution without an overwhelming amount of wires (Supplementary Material Video 4). State-of-art regarding applications adopting FBGs as transducers provide evidence of tactile sensors used in different scenarios. Compared to related works (i.e., soft tactile sensors embedding FBGs) (Heo et al., 2006; Saccomandi et al., 2015; Song et al., 2015; Jiang and Xiang, 2017; Negri et al., 2017; Li et al., 2018; Pedroso et al., 2018), the present sensor shares the concept of encapsulating the optical fiber in a soft matrix. Such cover not only protects the fiber from mechanical ruptures but also affects the transduction of the signal by mediating the transmission of pressure to the buried FBGs. In comparison to previous studies embedding FBGs in prototypical matrices (e.g., parallelogram bricks), the location of our FBGs were based on the design of the robotic hand, expressly functional to gripping tasks. One common elastomer used as encapsulating material is PDMS (Heo et al., 2006; Saccomandi et al., 2015; Song et al., 2015; Jiang and Xiang, 2017), while in this study a soft Dragon Skin silicone (20 medium, Smooth-on, USA) was used, due to its higher flexibility and lower delamination between layers. Further details on the adopted elastomer, are given in the Materials and Methods section.

The scope of the present study goes beyond the development of a soft and flexible tactile sensor. The novelty of the work also relies in the demonstration of a closed-loop control strategy for fine manipulation (Fragile Task), and in extracting features of manipulated objects, whereas in state of the art studies FBG wavelength variations were used to estimate several quantities (e.g., pressure, force, hardness) but within an open-loop scheme, without affecting the control variable. The work is organized as follows: Section Materials and Methods describes the transduction principle of the FBG transducers as well as the Cam-Hand design, the fabrication process and the control system. Furthermore, the same section reports about the experimental protocols and the data analysis. Results are presented in section Results, followed by the discussion and conclusion in section Discussions and Conclusions.

## Materials and Methods

### Fiber Bragg Grating Transduction Principle

An FBG is a reflector, formed by systematic variation of refractive index, inscribed in the core of an optical fiber. This resonant microstructure acts as a narrow band filter. When light propagates along the optical fiber, and reaches the etched FBGs, part of the source is reflected. This reflected signal is called Bragg Wavelength (λ_B_) and it depends on the grating spatial period (Δ_B_) and the effective refraction index (η_eff_) of the optical fiber as in Equation (1):

(1) $$\begin{array}{l} \lambda_{\text{B}}=2\cdot \eta_{\text{eff}}\cdot \Lambda_{\text{B}} \end{array}$$

Strain conditions and temperature variations imparted on the FBGs lead to variation of λ_B_ resulting in changes of the grating spatial period (Λ_B_), or effective refraction index (η_eff_). In the present work the contribution of temperature is negligible, since the whole experimental session was performed at room temperature.

### Sensorized Robotic Hand Design

The Cam-Hand body houses the driving electronics and three brushed DC motors (Maxon precision motor, Sachseln, Switzerland). The finger geometry follows a cam profile on the outside and a hook style shape on the inner profile. The system is comprised of two outer fingers slaved together and two inner fingers that are independent. Through continuous rotation of the fingers the Cam-Hand is able to achieve a huge number of configurations and grasping angles.

The Cam-Hand includes four fingers and the inner fingers were sensorized due to their independent actuation. Optical fibers (Technica Optical Components, Atlanta, GA, USA) that exhibit a diameter of 80 μm (100 μm with polyimide coating) were chosen as small diameters allow for low bending radius configurations. The fibers house 6 FBGs, each grating is 8 mm long and located at a distance of 10 mm, center-to-center, from the adjacent FBG. Table 1 provides further details about the chosen technology. The optical fibers were encapsulated in a soft polymeric material integrated into the rigid artificial finger. According to previous works (Massari et al., 2018), Dragon Skin (20 medium, Smooth-on, USA) was chosen as soft material for encapsulating the optical fibers. This polymer shows remarkable physical properties such as high elongation at break and high flexibility (Cai et al., 2013). Moreover, during grasping, silicone mediated the transmission of pressure to the buried FBGs, applied by the grasped object to the robotic finger. Maintaining the same design of the previous Cam-Hand, new customized fingers were developed, in aluminum, with a notch to allow the insertion of the soft material and the relative optical fibers (Figure 1B). Such a groove held an irregular shape that followed the curvature of the robotic fingers. Both sides of the finger presented the groove and were connected by means of a series of holes (3.5 mm diameter) whose purpose was to hold the polymer in a fixed position. Approximately, the notch resulted 62 mm in length, 4.4 mm in width and 2.5 mm in height (Supplementary Figure 1). The liquid polymer was casted to fill the notch and thus filling out the shape of the artificial finger when not sensorized. The final design includes two optical fibers located at each side of the finger. The sensitive area of the finger is approximately 60 mm, which corresponds to the front part of the finger, namely the area responsible for the grip. Several iterations of molds were created to realize the polymeric filling and fabrication process. This involves three consecutive steps:

- Development of the first layer of silicone with a groove to insert the optical fiber
- Insertion of the optical fiber in the right position
- Development of the second layer of silicone to cover and protect the optical fiber

**Table 1:** Datasheet of the optical fibers integrating FBG transducers.

**Reflectivity**	**Coating**	**Wavelengths**	SLSR^*^	FWHM^**^
**OPTICAL FIBER SPECIFICATION**				
>70%	Polyimide	1535:5:1570nm	>15db	0.5nm

* *SLSR, Side Lobe Suppression Ratio*.

** *FWHM, Full Width Half Max*.

In step (i) and (iii) silicone was degassed to minimize air bubbles and cured at room temperature until solidification was reached.

### Cam-Hand Controller

**Figure 2:**
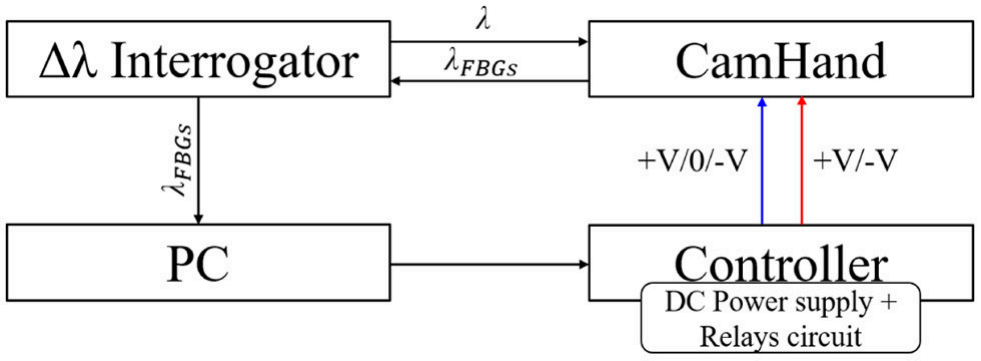
Block diagram of the experimental setup. The blue line shows the static controller while the red line shows the dynamic controller.

The movements of the Cam-Hand were piloted by means of a DC voltage supply (HMC804x Power Supply, Rohde & Schwarz, Munich, Germany), a relays circuit (4-channel 5V USB Relay Module, SainSmart, USA) and an optical interrogator (Hyperion si155, Micron Optics, GA, USA), that was reading the FBGs output. A Graphical User Interface was realized in LabVIEW (National Instruments, TX, USA) to control the previous units and for data acquisition (Figure 2). A positive voltage was applied to the DC motor to close the hand and vice versa a negative voltage applied to open it. At constant load, higher voltage values entailed higher motor speed (*rpm*) and consequentially faster movements of the fingers. Through the power supply, the voltage flow was regulated to set the velocity and the relays were switched on/off to close/stop/open (+V/0/-V) the fingers. The initial configuration, also called *free configuration* corresponded to a condition where the fingers were open and ready to perform the grasp, while the *grasp configuration* matched with the condition of the fingers closed around the objects. Two controllers were developed: (i) static controller and (ii) dynamic controller. In the first case a fixed voltage equal to 13.5 V was given to the motor, thus allowing the fingers to close or open at constant speed. Depending on the FBGs output and through switching the relays, the static controller achieved the action of closing, stopping and opening the fingers. Two thresholds, lower and upper, were set on the mean of all FBGs wavelength variation. Enabling the static controller to activate the transition from the *free configuration* to the *grasp configuration*. When the mean wavelength variation was lower than the first threshold, the controller allowed the flow of a positive voltage and the corresponding closing movement. When the mean wavelength variation trespassed such threshold, due to the higher pressure applied from the object to the sensorized finger and the consequentially higher strain suffered by the optical fiber, and entered in the *grasp configuration*, the controller disabled the voltage flow and stopped the hand. Opening the hand, thus giving negative voltage, took place when the mean wavelength variation raised over the upper threshold. In the second controller, instead, the given voltage was not constant but function of the mean wavelength variation. Through a PID controller (Proportional—Integrative—Derivative), in a closed loop, different values of voltage (based on the mean wavelength variation) were given to maintain a steady grab condition. A desirable value was established for the mean wavelength variation, corresponding to a certain grab condition, hereafter called set point. The controller was aimed at regulating the voltage values and switching the relays on/off in order to reach this value and sequentially to maintain it. Scope of such controller was to respond, dynamically, with different voltages to changes in size of the grasped object without slipping or breaking (Supplementary Material Video 3).

### Experimental Materials and Protocols

**Figure 3:**
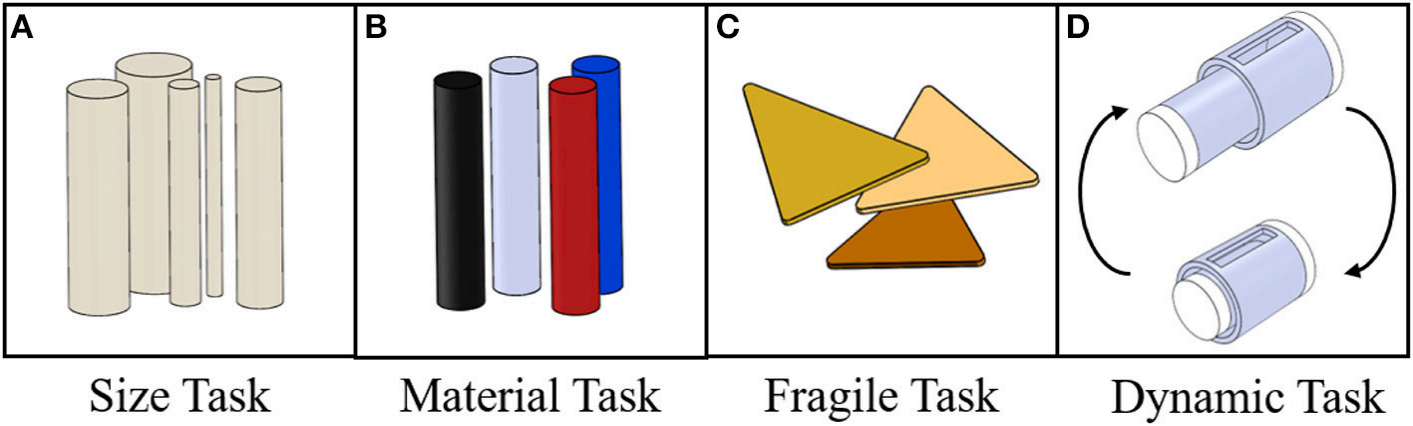
Representation of the used objects for the different tasks. **(A)** Material task: 5 ABS 3D printed cylinders with increasing diameter ranging from 10 to 50 mm with 10 mm step. **(B)** Material task: 4 cylinders with fixed diameter of 30mm, but with different and increasing Young Modulus ranging from ≈200 kPa to ≈70 GPa. **(C)** Fragile task: Nachos used to test the sensitivity of the Cam-Hand. **(D)** Dynamic task: mechanical jack with variable length.

The performance of the proposed version of the Cam-Hand was evaluated through different tasks that involved the action of grabbing several objects in various conditions (Supplementary Figure 2). Within this work, four tasks were performed: (i) Size Task, (ii) Material Task, (iii) Fragile Task, and (iv) Dynamic Task. The first and second tasks assessed the capability of the sensorized fingers to estimate mechanical properties of grasped object, namely size and rigidity. The third task, representing a qualitative test, evaluated the ability of the system to grab fragile objects without slipping or breaking them, thus obtaining a measure of the sensitivity of the Cam-Hand (Supplementary Material Videos 1, 2). The last task measured the Cam-Hand capacity to dynamically adapt its position based on objects that could change size (Supplementary Figure 3). For the Size Task 5 plastic cylinders, 3D printed in ABS, with varying diameter from 10 mm to 50 mm with step of 10 mm were realized. The height of such cylinders was constant and equal to 150 mm (Figure 3A). For the Material Task 4 cylinders, 30 mm in diameter and of 150 mm height, realized in different materials were used. Such cylinders had increasing Young Modulus: Dragon Skin E ≈ 0.34 MPa, Vytaflex E ≈ 2 MPa (60 A, Smooth-on, USA), ABS E ≈ 2.2 GPa and Aluminum E ≈ 70GPa (Figure 3B). For the Fragile Task commercial nachos were used, that can be considered to be very fragile objects (Figure 3C). The Dynamic task involved the realization of a mechanical jack composed by two concentric cylinders, 29 mm and 23 mm in diameter and 40 mm and 28 mm in height, respectively. These objects were realized in ABS with a 3D printer and the top and bottom part were covered by a thin layer (5 mm) of Dragon Skin 20. The mechanical jack was attached to a motorized linear stage (A-LST0500B-C, Zaber Technologies Inc., Vancouver, Canada) that could control its length by moving backwards and forwards (Figure 3D).

**Figure 4:**
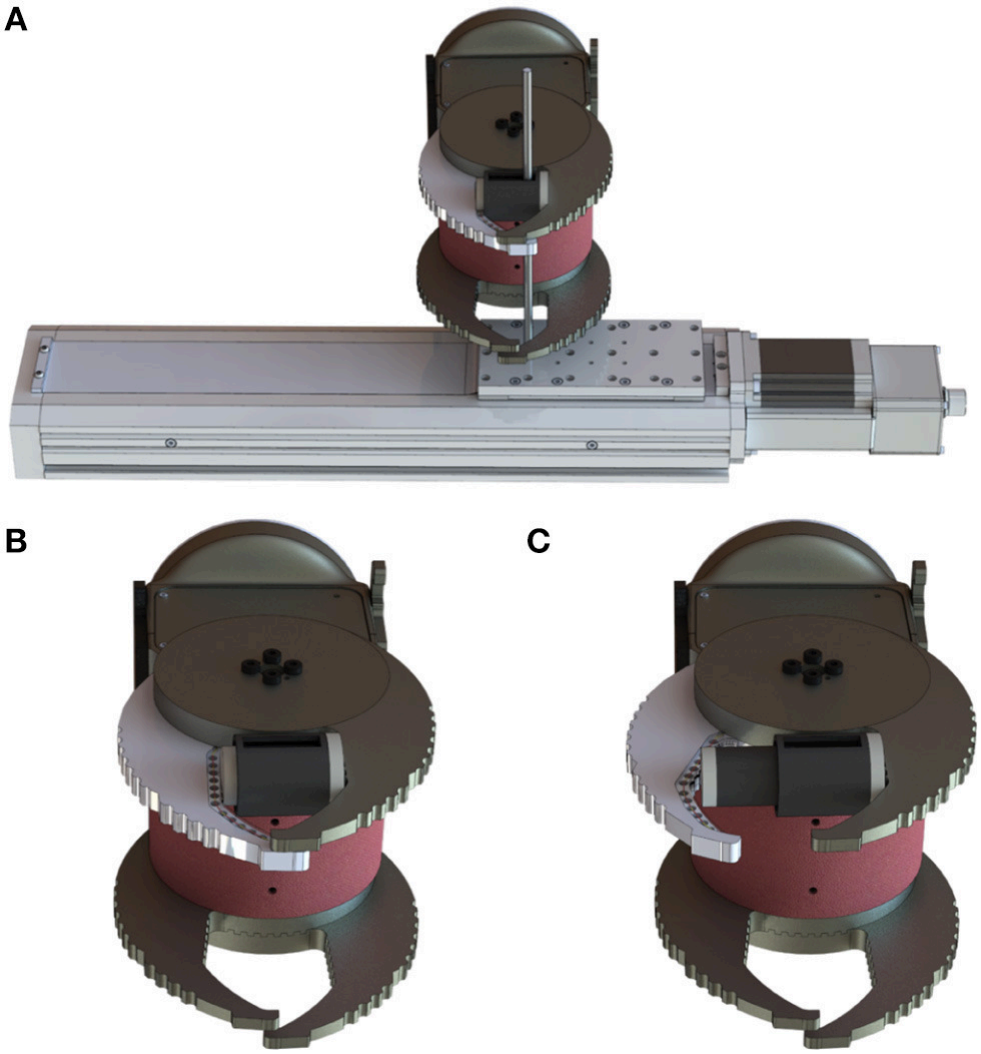
**(A)** Experimental setup for the dynamic task. **(B)** Cam-Hand with mechanical jack at minimum extension. **(C)** Cam-Hand with mechanical jack at maximum extension.

For the first three tasks the procedure consisted of performing a grasp with the Cam-Hand piloted by the static controller. The sample was manually held between the robotic fingers in *free configuration*. Then, by enabling voltage flow, the Cam-Hand started closing its fingers until reaching the *grasp configuration*. After a few seconds of grasping, the robotic hand was manually released and brought back to the *free configuration*. Each trial of the first two tasks was executed 10 times for repeatability, thus having 50 tests for Size Task and 40 tests for Material Task. For the Fragile Task, 20 repetitions were achieved. For the first and the second task, the lower threshold was set to 0.01 nm, while for the Fragile Task it was set to 0.16 nm. In the Dynamic Task a grasp of the mechanical jack was performed with the Cam-Hand piloted by the dynamic controller. The set-point was set to 0.16 nm, this value was reached after enabling voltage flow and passing from *free configuration* to *grasp configuration*. After the grasping action, random values of travel range (from −25 to 25 mm) and velocity (from 0.5 to 3 mm/s) were given to the linear motorized stage. Consequentially, the mechanical jack linked to the stage started to move with different random velocity into a different random position. The Cam-Hand adapted its position, with a velocity proportional to the velocity of the stage, based on the mean wavelength variation (Figure 4). Moreover, another test was performed in which random values of travel range were given, but the values of velocity were increasing, starting from 0.5 mm/s and increasing by 0.1 mm/s each 0.75 s. After reaching the destination the velocity was reinitialised to 0.5 mm/s. To measure the force required to break the commercial nachos, an experimental task was performed. Fifteen nachos were brought to fracture by applying a compressing force using a robotic platform composed by a load cell (Nano 43, ATI Industrial Automation, Apex, NC, USA) and a motorized vertical stage (8MVT120-25-4247, STANDA, Vilnius, Lithuania). During the experiments, the motorized stage was commanded with a speed of 2.5 mm/s until breakage of the nachos samples was achieved, while the load cell was tracking the applied load force. We thus estimated the sensitivity of the FBG sensor as the ratio between the peak wavelength variation measured during grasping and the load breaking the nachos.

### Data Analysis

The Neural Network Pattern Recognition App, developed within the Neural Network Toolbox in Matlab (MathWorks, Inc., MA, USA) was employed to predict the diameter and the rigidity of the grasped objects from the FBGs wavelength variation, in the Size Task and in the Material Task, respectively. In the Size Task, the proposed classifier comprised 12 input neurons, namely all the FBGs reflected the wavelength exerted during the grab, while in the Material Task there was 1 input neuron, namely the slope of the wavelength variation function of time (Δλ/Δ*t*) tracked during the grab. Both the neural network comprised 10 hidden neurons and 1 output neuron, which was the cylinder diameter value in mm in the first task and the material rigidity in the second task. The neural networks were trained using the conjugate gradient backpropagation method. The experimental data were divided into three complementary subsets: (i) training set, (ii) validation set, and (iii) testing set. For each class (i.e., diameter), 10 repetitions were performed and 8 trials were used for training, 1 trial for validation and 1 trial for test. To reduce variability, multiple rounds of cross-validation, using different partitions, were performed. The “Leave-one-out cross-validation” method was adopted, which used one observation as a test set (and one as a validation set) and the remaining as training set. This partition was repeated, each time changing the test set and consequentially the other two subsets, until all the 10 trials were considered one time as test set. The results of the different cross-validation were combined (i.e., averaged) to assess the neural network's predictive performance by means of a confusion matrix. Within the Fragile Task, the ability of the Cam-Hand to deal with fragile objects was assessed by calculating the number of broken samples during grasps. To assess the performance of the Cam-Hand to follow the changes in the grasped objects (Dynamic Task) the Root Mean Square Error (RMSE), the Normalized RMSE (NRMSE) and the NMRSE calculated for the data included in the interquartile range (NRMSE_(IQR)_) were calculated as expressed in Equations (2–4).

(2) $$\begin{array}{l} \text{RMSE}=\sqrt{\frac{\sum_{n=1}^{N}{\left(x_{1,n}-x_{2,n}\right)}^{2}}{N}} \end{array}$$

(3) $$\begin{array}{l} \text{NRMSE}=\text{RMSE}/\bar{x} \end{array}$$

(4) $$\begin{array}{l} {\text{NRMSE}}_{\left(\text{IQR}\right)}={\text{RMSE}}_{\left(\text{IQR}\right)}/{\bar{x}}_{\left(\text{IQR}\right)} \end{array}$$

## Results

**Figure 5:**
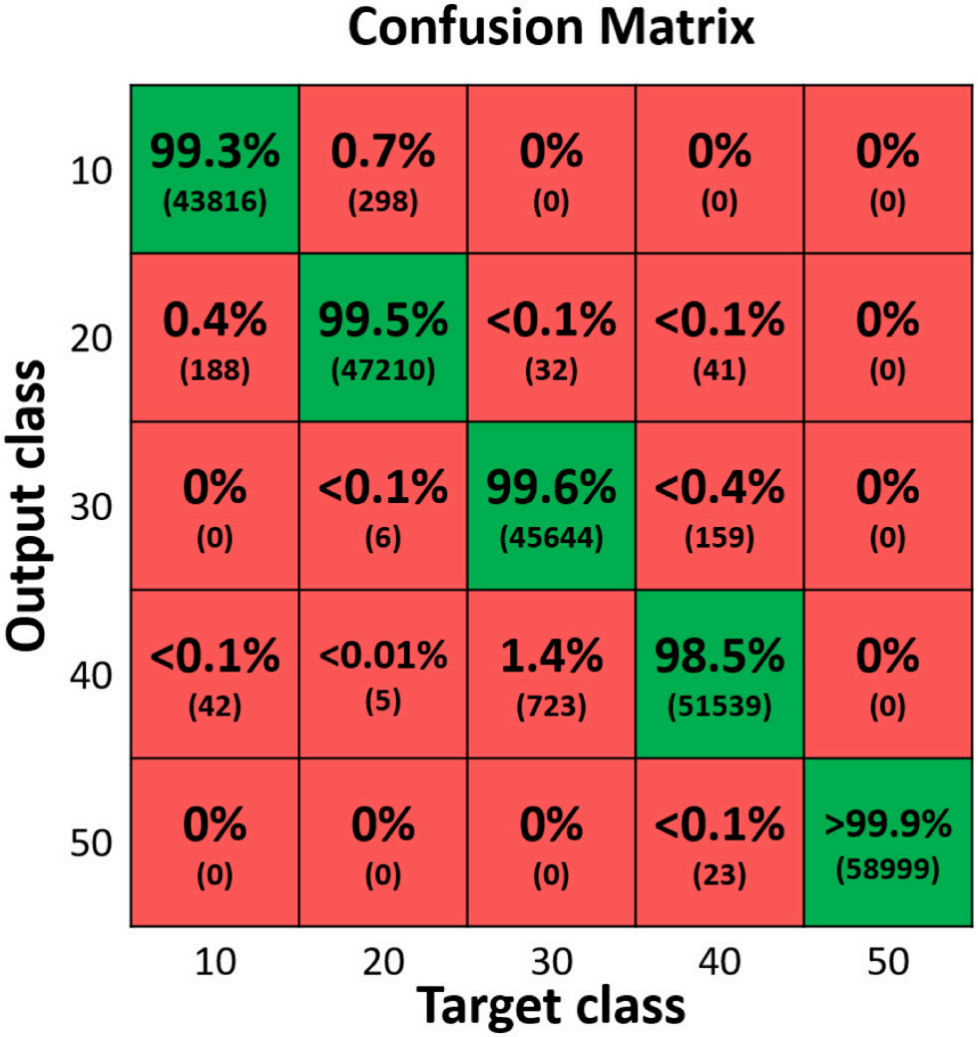
Confusion matrix showing the accuracy (99.36%) of a classifier for size discrimination of the grabbed sample. 5 cylinders with different size were tested. ten trials were performed per each sample. Eight out of 10 trials were used for training, 1 out of 10 for validation and 1 out of 10 for testing. To improve generalization we applied the “leave one out cross validation” method. The numbers in brackets represent the experimental data processed by the classifier.

**Figure 6:**
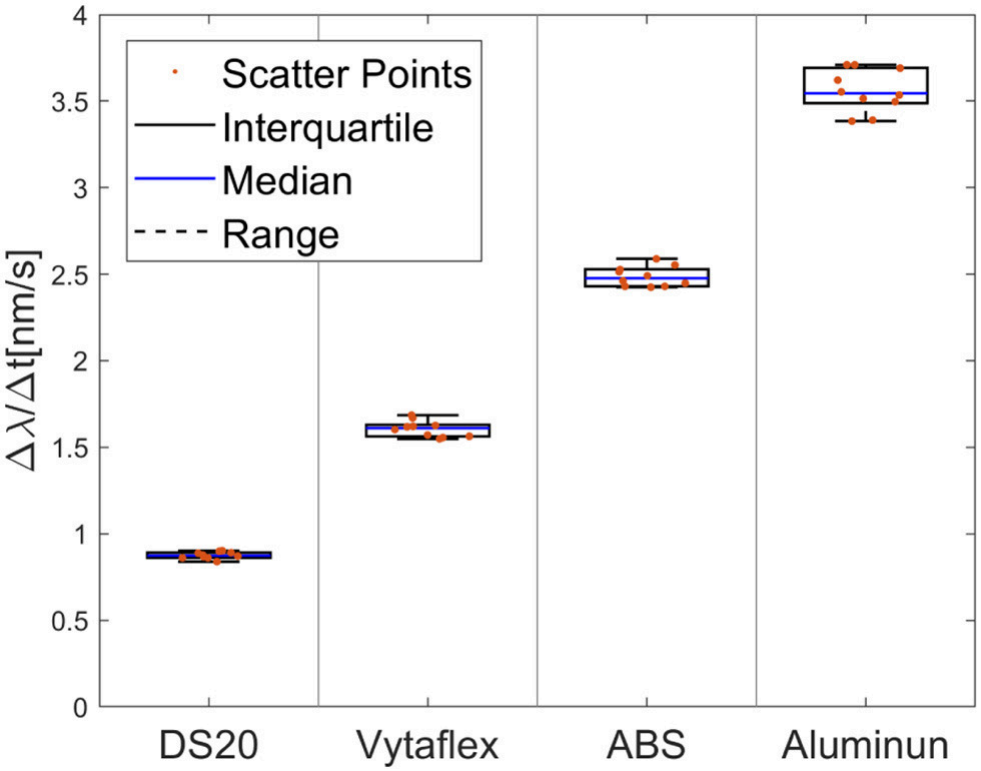
Box plot of the FBGs Δλ/Δ*t* (slope of the signal) for the different materials. From left to right the Young Modulus is increasing. Boxes represent interquartile ranges; blue lines show the median value and black dashed lines show the complete range across samples.

**Figure 7:**
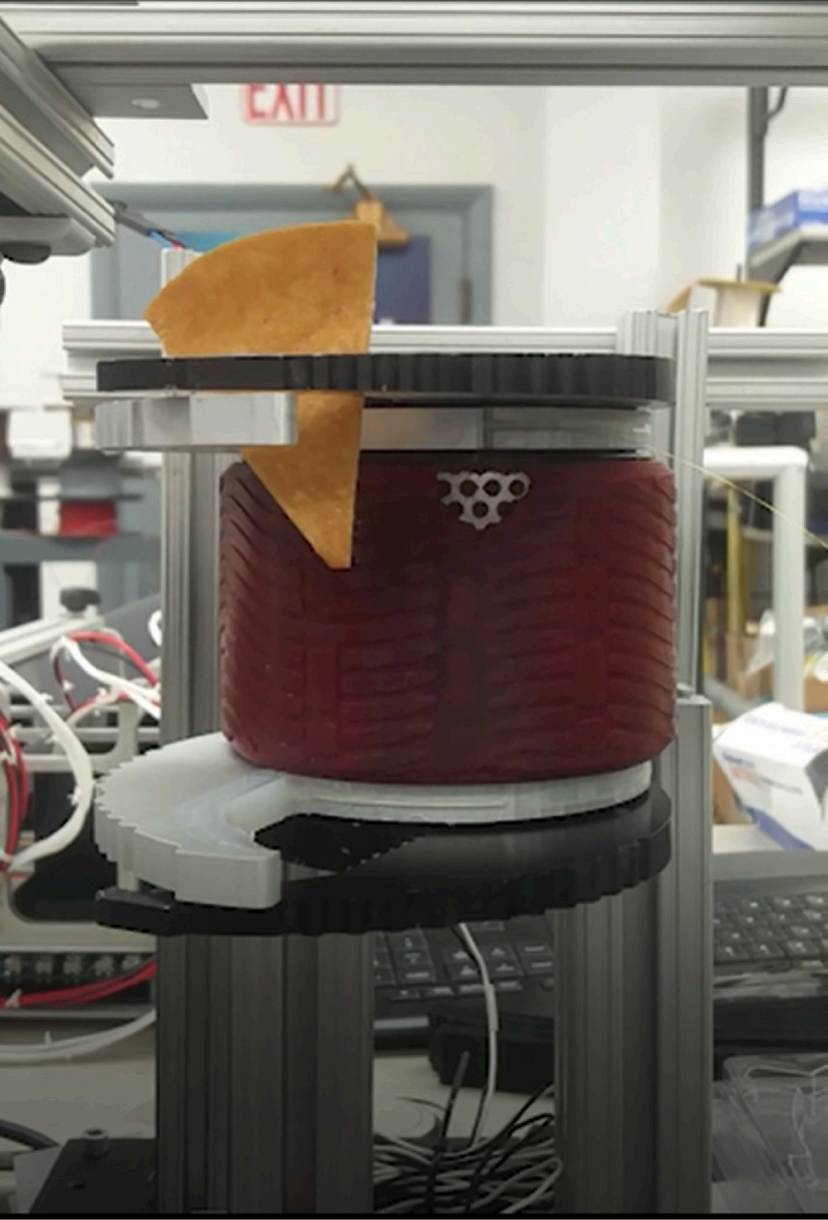
Picture showing the fragile test performed by grasping a commercial nacho.

**Figure 8:**
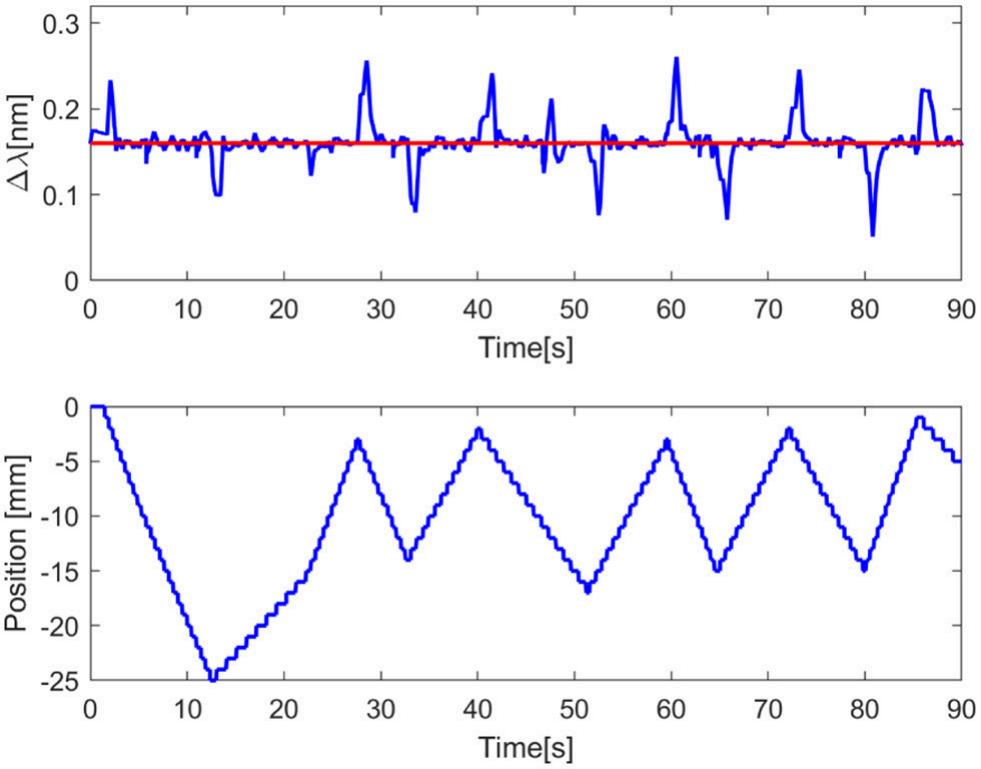
Graph showing the dynamic task results. In the upper plot, the red line represents the set point value equal to 0.16 nm, the blue line represents the process variable, namely the wavelength variation of the mean of the FBGs. In the bottom plot the blue line represents the position of the motorized translational stage.

**Figure 9:**
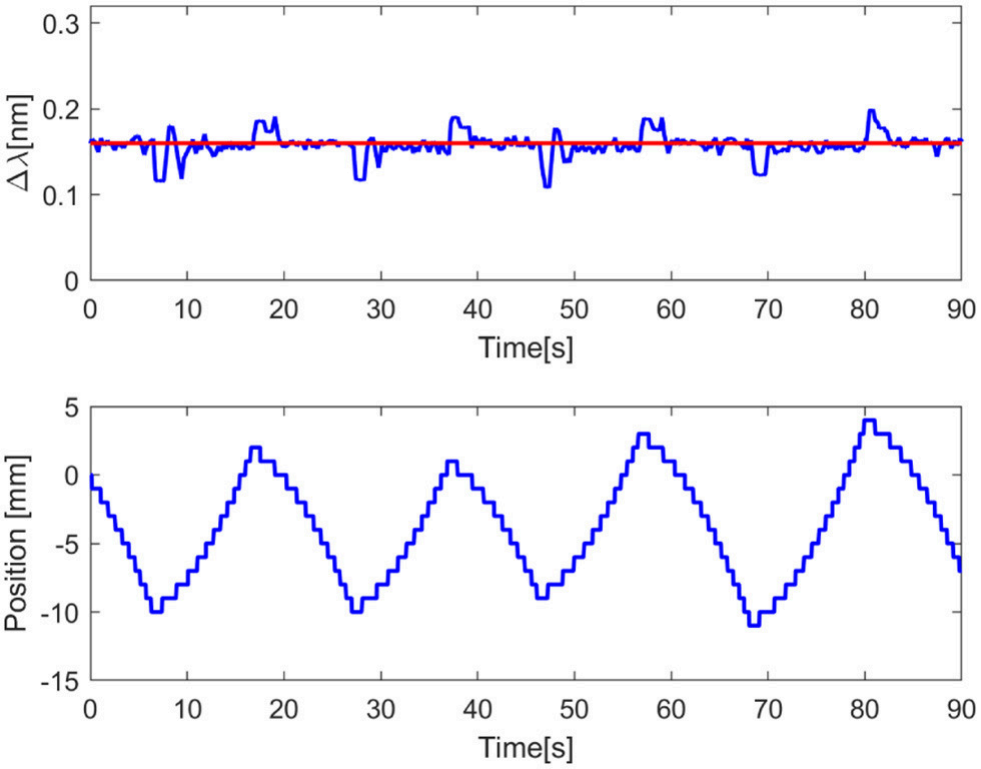
Graph showing the dynamic task results. In the upper plot, the red line represents the set point value equal to 0.16 nm, the blue line represents the process variable, namely the wavelength variation of the mean of the FBGs. In the bottom plot the blue line represents the position of the motorized translational stage.

Through different tasks, the capability of the proposed FBGs-based robotic hand was assessed to provide tactile feedback. By evaluating the performance of the proposed classifier for size recognition of different grasped objects, an overall accuracy of 99.36% was achieved. Individual accuracy values were calculated for each diameter: 99.3% for 10 mm, 99.4% for 20 mm, 99.6% for 30 mm, 98.5% for 40 mm and 99.9% for 50 mm. Moreover, it is relevant to observe that misclassification, within the different classes, happened mainly with their relative neighbors (Figure 5). Physical properties of grasped objects strongly affected the FBGs wavelength variation. The slope of the wavelength variation function of time (Δλ/Δ*t*) increased monotonically with increasing Young Modulus across the different materials (Figure 6). High repeatability was achieved across all trials: the median value of the slope was 0.87 ± 0.02 nm/s for Dragon Skin 20, 1.61 ± 0.04 nm/s for Vytaflex, 2.48 ± 0.05 nm/s for ABS and 3.54 ± 0.12 nm/s for Aluminum (Figure 6). Furthermore, the classifier used to predict the rigidity showed an accuracy of 100% for all the classes. Within the Fragile Task, when performing a grasp on the nachos, only one sample among the 20 executed trials was broken (Figure 7). The results of the Dynamic Task presented similar performances in the two experimental conditions, both in the experiments with constant velocity (Figure 8) and in those where velocity increased from one position to the next (Figure 9). Error values were: RMSE = 0.019 nm, NRMSE = 12% and NMRSE_(IQR)_ = 1.2% for the first condition and RMSE = 0.014 nm, NMRSE = 9% and NMRSE_(IQR)_ = 2.2% for the second condition. The mean force value needed to break the sample was experimentally estimated to be 9.49 N ± 3.13 N. Combining this result with the FBG wavelength variations recorded in the fragile task turns out in a sensitivity estimation of at least 139 pm/N.

## Discussions and Conclusions

The results obtained through the different tasks had very high precision in identifying relevant properties of grasped objects (Size Task, Material Task and Fragile Task) as well as the contact conditions (Dynamic Task). Within the Size Task, the sensorized hand allowed recognition of the diameters of the cylinders from Bragg wavelength variations, using the proposed neural network for pattern recognition (99.36% accuracy). Since the trials of the Material Task were performed using the static controller, thus selecting a constant velocity for fingers movements, such condition also allowed for estimation of the hardness of the different materials via the temporal variation of the Bragg wavelength (100% accuracy using the proposed classifier). Our hypothesis relied on the evidence that, at constant speed, harder material involved faster Bragg wavelength signal variation. Within the Fragile task, observations made on several grabbed samples (nachos) allowed for the reliability of the sensorized finger to be evaluated in handling such kind of fragile objects. The achieved experimental results are quite generalized, since each nacho had a different shape and size. The scope of the task was to understand qualitatively the sensitivity of the sensor. The results of the Dynamic Task provided evidence for the capability of the sensorized finger to adapt its position based on the variation of the length of a mechanical jack. Furthermore, it is clear that in both the performed conditions and during the entire travel range, the difference between the set point and the process variable was very low as demonstrated from the NRMSE_(IQR)_ values. However, the results presented some peaks related to the phase of inversion of the motion of the stage, as highlighted from the NRMSE values, which were based on the entire raw dataset and not only the interquartile range. When the stage reached a position, it moved immediately into another position and therefore direction. Consequentially, the robotic finger passed from the action of closing to the action of opening (or vice versa) that caused an error of the controller in maintaining constant grasp conditions. Although the PID controller was not always accurate, the maximum and minimum values of wavelength variation were acceptable for keeping a good grasp without breaking or slipping the object. We believe that the peaks encountered in the task are not related to the sensor performance but instead related to the used motor drivers (relays circuit). The scope of the work was mainly centered around the evaluation of the proposed tactile sensorization and not around the realization of a perfect controller. Furthermore, the mechanical jack linked to the motorized stage through a long steel bar could have influenced the presented results. Future work will address the integration of the sensorized Cam-Hand in a robotic arm, thus bypassing the issues of the controller, since the actuation part will be managed by the motor controllers of the arm. Moreover, further investigations will carry out experimental tasks with other shapes (not only cylinders) but will also evaluate wider ranges of diameters and rigidity to increase the variety of the grasped samples. Further studies will also deal with the calibration of the system in order to estimate the relationship between the wavelength variation and the applied pressure to the robotic finger. A limitation of the present study is related to temperature compensation ability. FBGs directly respond to strain and temperature changes. Such intrinsic sensitivity to both physical variables requires a compensation method to split the contribution of mechanical actions from the contributions of possible temperature changes. Considering that the environmental conditions of the laboratory were stable within the performed experiments, the temperature contribution was not considered. Future studies will aim at introducing temperature compensation solutions, for example by means of dummy FBGs not being affected by strain but by temperature changes only.

The present paper introduced a robotic hand sensorized with optical fibers, embedding FBGs transducers, to convey tactile feedback in robotic manipulative tasks. To the best of our knowledge this is the first study that demonstrates the application of FBG technology in a robotic hand, in order to achieve fine object manipulation and features extraction based on closed-loop control. The choice to sensorize such a gripper with optical fibers is based on their flexibility in the integration process, but also on their high sensitivity in strain measurements. Thanks to this integration of tactile sensors, the new Cam-Hand design targeted the following abilities: (i) estimation of the grasped object size, (ii) detection of a value related to the Young modulus of the grasped objects (iii) grasping of objects with different mechanical properties (i.e., fragile, deformable, stiff) without their slippage or breakage, and (iv) dynamic adaptation of the fingers in order to maintain constant wavelength variation, independent of the shape of the objects. We believe that multiple advantages of FBG technology, demonstrated throughout the paper, can move forward the current state of the art. Beyond the aforementioned advantages, optical fibers ensure light weight solutions and distributed sensor capabilities. Another interesting advantage of using such a technology is the multiplexing capability. Multiple FBGs can be housed along one single optical fiber by means of just minor arrangements, hence improving the sensing capabilities without drawbacks in terms of complexity and bulkiness. Finally, FBGs pave the way for RoboSimian to operate in those application scenarios that require electromagnetic immunity, where most of the conventional sensors are unsuitable.

## Author Contributions

LM, CO, and KC conceived the sensorized hand. LM and KC realized the sensorized hand. ES supported the design by means of engineering tools and contributed to data interpretation. LM, CO, ES, and KC designed the experimental protocols. LM, RD, and KC performed the experiments. LM, RD, and JB analyzed the data. LM, CO, ES, and KC discussed the results. LM, CO, and KC wrote the paper. CO and KC supervised the study.

### Conflict of Interest Statement

The authors declare that the research was conducted in the absence of any commercial or financial relationships that could be construed as a potential conflict of interest.
