# Supplementary material for: Tactile Sensing and Control of Robotic Manipulator Integrating Fiber Bragg Grating Strain-Sensor
Source: Front Neurorobot. 2019 Apr 5;13:8. doi: 10.3389/fnbot.2019.00008 (PMC6477702; doi:10.3389/fnbot.2019.00008)
Supplement: Supplementary file 5 [file Data_Sheet_1.docx]

Supplementary Material

Tactile Sensing and Control of Robotic Manipulator Integrating Fiber Bragg Grating Strain-Sensor

Luca Massari^1*^, Calogero M. Oddo^1^, Edoardo Sinibaldi^2^, Detry Renaud^3^, Joseph Bowkett^4^, Kalind C. Carpenter^3*^

^1^The BioRobotics Institute, Polo Sant’Anna Valdera, Scuola Superiore Sant’Anna, Viale Rinaldo Piaggio 34, 56025 Pontedera, Italy

^2^Center for Micro-BioRobotics, Istituto Italiano di Tecnologia, Viale Rinaldo Piaggio 34, 56025 Pontedera, Italy

^3^Jet Propulsion Laboratory, California Institute of Technology, NASA, 4800 Oak Grove Drive, 91109 Pasadena, California, USA

^4^Department of Mechanical & Civil Engineering, California Institute of Technology, 1200 East California Boulevard, 91125 Pasadena, California, USA

*** Correspondence:**Luca Massari - Email: [luca.massari@santannapisa.it](mailto:luca.massari@santannapisa.it)

Kalind C. Carpenter - Email: [kalind.c.carpenter@jpl.nasa.gov](mailto:kalind.c.carpenter@jpl.nasa.gov)


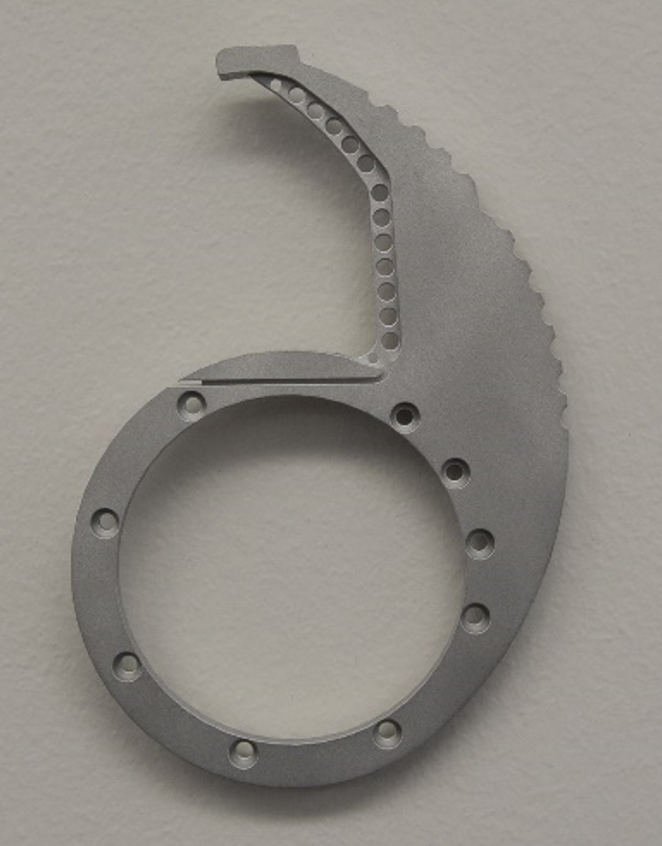


**Supplementary Figure 1.** Cam-Hand finger before the integration of the soft polymer embedding the optical fiber. In the figure it is highlighted the groove for the insertion of the Dragon Skin 20 silicon.


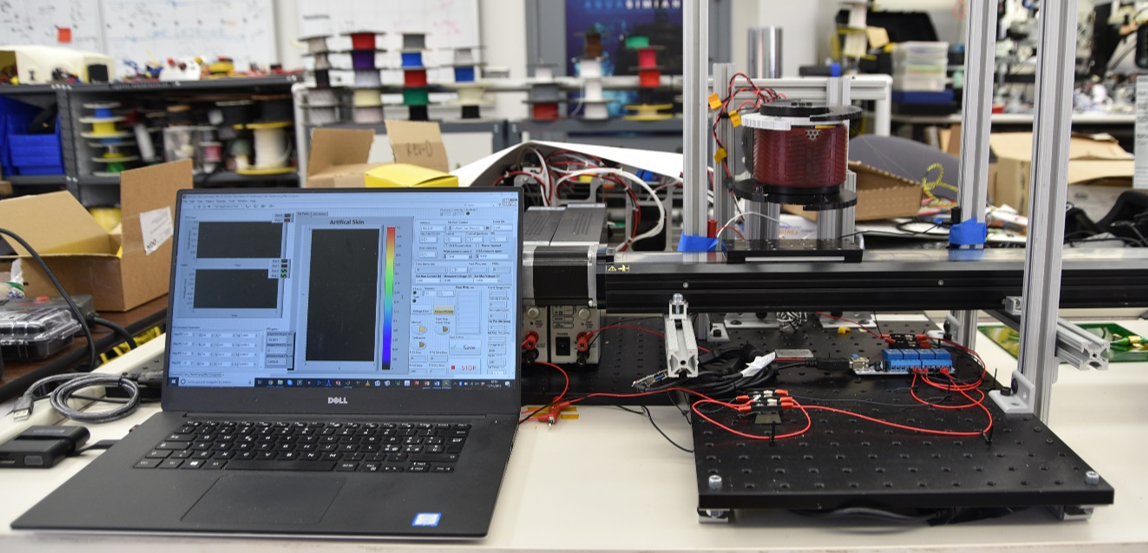


Supplementary Figure 2. Experimental setup for assessing the performance of the sensorized finger.


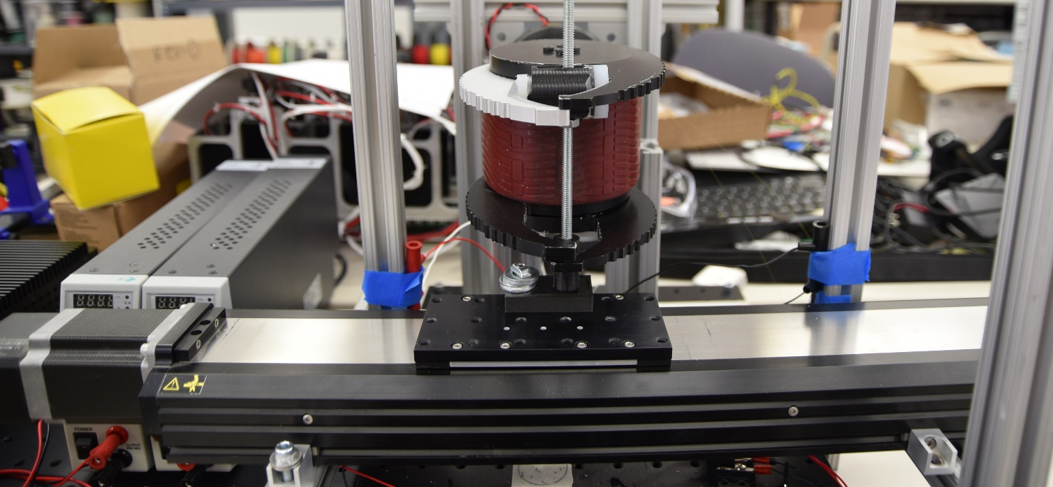


Supplementary Figure 3. Experimental setup during the Dynamic Task
